# Assessment of the speed of transmission of *Ehrlichia canis*, *Anaplasma phagocytophilum*, and *Borrelia burgdorferi* sensu stricto by infected ticks through an in vitro experimental model

**DOI:** 10.1186/s13071-025-06798-9

**Published:** 2025-05-20

**Authors:** F. Beugnet, M. Madder, A. Joubert, I. Bouzaidi Cheikhi, M. Chajia, J. F. Besselaar, D. Y. Tan

**Affiliations:** 1 Boehringer Ingelheim Animal Health, 29 Av. Tony Garnier, Lyon, France; 2 Clinvet, B.P., 301, 28815 Mohammedia, Morocco; 3 Clinomics, Universitas, PO Box 11186, Bloemfontein, 9321 South Africa; 4 Clindata, Itec Building, 14 CP Hoogenhout Street, Langenhoven Park, Bloemfontein, 9301 South Africa

**Keywords:** Tick-borne pathogens, Speed of transmission, *Ehrlichia canis*, *Anaplasma phagocytophilum*, *Borrelia burgdorferi* s.s., In vitro feeding system, Canine

## Abstract

**Background:**

Canine vector-borne diseases (CVBDs) have significant clinical and public health implications.

**Methods:**

This experimental study used a validated continuous-flow in vitro feeding system (CFIFS) to investigate the speed of transmission (SOT) of three tick-borne pathogens (TBPs): *Ehrlichia canis* by laboratory-infected *Rhipicephalus sanguineus* (18.3% infection rate), *Anaplasma phagocytophilum* by laboratory-infected *Ixodes ricinus* (56%), and *Borrelia burgdorferi* sensu stricto (s.s.) by laboratory-infected *I. ricinus* (76%). Three experiments were conducted, one per pathogen/tick model. A total of 58–60 ticks were used per feeding system. Four to six replicates were obtained per experiment. All ticks were laboratory-reared. The tick infections were performed by feeding the nymphal stages on infected hosts.

**Results:**

All ticks began to attach and feed 3 h after being introduced to the feeding system. At the maximum attachment, 89.7% of *R. sanguineus* were attached at 57 h, with 4–30% attachment at 51 h for *I. ricinus* infected with *A. phagocytophilum*, and 6.3–47.9% at 48 h for *I. ricinus* infected with *B. burgdorferi* s.s. Polymerase chain reaction (PCR) tests were used to detect the presence of pathogens from blood samples collected every 3 h. Swab samples from the inner face of the feeding membrane were also collected and tested every 6 h during the *B. burgdorferi* s.s. study. In this experimental in vitro design, after the first tick attachments were observed, *E. canis* exhibited SOT of 3–6 h, *A. phagocytophilum* of 12–15 h, and *B. burgdorferi* of 42–45 h in blood but only 3–6 h on inner membrane swabs.

**Conclusions:**

The findings of this in vitro study highlight the transmission time of some TBPs, confirming previous data obtained in vitro or in vivo, by using the same design for all tick/pathogen models. This is a way to estimate the possibility of using acaricidal drugs to block pathogen transmission based on the SOT and the speed of kill of these compounds.

**Graphical Abstract:**

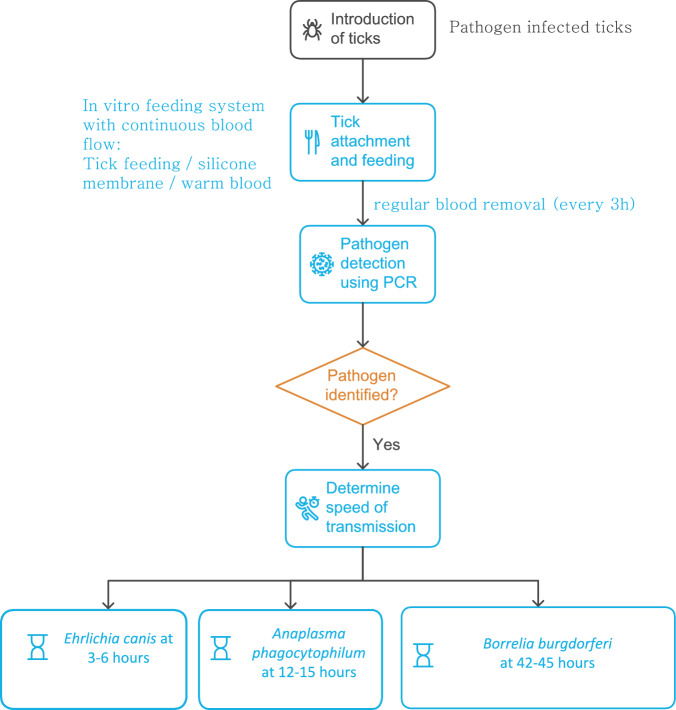

## Background

Tick-borne diseases represent a significant public health and veterinary concern, impacting both humans and animals. Numerous tick species are capable of transmitting a variety of pathogens to their host during their blood meal, including bacteria, viruses, and protozoa, causing life-threatening illnesses [1].

The tick species of the family Ixodidae are primarily responsible for spreading most tick-borne pathogens (TBPs) of veterinary and/or public health relevance, including *Ehrlichia canis*, *Anaplasma phagocytophilum*, and *Borrelia burgdorferi*.

*Ehrlichia canis* is a TBP that causes canine monocytic ehrlichiosis and is transmitted by the brown dog tick, *Rhipicephalus sanguineus* sensu lato (s.l.). The main, and probably the only, vector for *E. canis* in Europe is the tick *R. sanguineus* s.l., known as the brown dog tick. This tick was shown experimentally to be a competent vector for *E. canis*, which is transmitted transstadially by *R. sanguineus* ticks [2]. An original study published in 2013 including dog infestation and in vitro testing showed that transmission of *E. canis* by *R. sanguineus* ticks started within 3 h after tick attachment to the dog [3]. Another tick species, *Dermacentor variabilis*, was shown to be a potential vector for *E. canis,* nevertheless, this demonstration has not been confirmed recently [4]. *R. sanguineus* is considered the major vector in the USA [5]. Whereas several species of *Ehrlichia* have been identified in dogs in the USA (i.e., *E. canis*, *E. ewingii*, *E. chaffeensis*, *E. muris eauclairensis*, and Panola Mountain *Ehrlichia*), *E. canis* remains the only one described in Europe [5, 6]. Dogs are the main reservoir, but other wild canids (foxes, wolves, jackals) can become infected. Moreover, several studies have reported the presence of *E. canis* DNA in cats and wild felids. However, experimental infection has only been reproduced in dogs [5, 6]. The disease is characterized by thrombocytopenia, leukopenia, fever, depression, and bleeding tendencies (epistaxis) [6, 7]. The zoonotic potential of *E. canis* has been mentioned, but no recent publications have confirmed this possibility [8].

*Anaplasma phagocytophilum*, a zoonotic TBP [9], causes canine granulocytic anaplasmosis and is transmitted in Europe by *Ixodes ricinus*. It is the causative agent of canine granulocytic anaplasmosis, a disease with non-specific clinical symptoms such as lethargy, reduced activity, fever, and inappetence [10–14]. *Anaplasma phagocytophilum* can also infect humans (causing human granulocytic ehrlichiosis) and several animals other than dogs, including cats, sheep, goats, cows, equines, rodents, roe deers, deers, and other wild mammals [15, 16]. From a clinical perspective, granulocytic anaplasmosis is characterized by a chronic fever, leukopenia, and thrombocytopenia [10, 12, 16]. Within *A. phagocytophilum* species, genetic variants have been identified and seem to be host-related [9, 16].

*Borrelia burgdorferi* s.l. is another zoonotic TBP [3] transmitted in Europe by *I. ricinus*. It causes Lyme borreliosis in humans, dogs, and horses [17]. Lyme borreliosis is the most common vector-borne disease observed in humans in Europe and the United States [17]. Infected dogs may develop fever, lameness, and polyarthritis. In humans, Lyme is a multisystemic disease that affects multiple organs, including the heart, joints, central nervous system, and brain. Symptoms include extreme fatigue, flu-like symptoms, arthritis, peripheral neuropathy, and cognitive dysfunction [17]. Lyme borreliosis is clinically less relevant in animals than in humans, and its prevalence is also low compared with other vector-borne diseases in dogs [18–20].

There is a gap in our current understanding of vector-borne pathogen (VBP) transmission times [20] ,as they are governed by a number of factors including tick vectors and pathogens, vector feeding behavior, and the susceptibility of the vertebrate host [20–23]. The knowledge of the dynamics of pathogen transmission has improved with the sensitivity of detection based on molecular biology. The first transmission times were studied through in vivo experiments, mainly using mice and infected nymphs. Eisen, in 2018, showed the transmission of *A. phagocytophilum* from *I. scapularis* nymphs to mice in less than 24 h [23]. The difficulty in conducting in vivo studies (i.e., ethical concerns, duration of studies, animal management considerations, etc.) led to the development of in vitro tick feeding systems. The original systems were based on tick feeding chambers [3, 17, 19, 24]. However, there were several replicates of chambers for each time point because the blood units were fixed [17, 19]. These feeding units were first developed by Guerin and Krober for *I. ricinus* in 2007 [24]. Understanding the speed of transmission (SOT) can aid in developing new prevention strategies against VBPs [25], in particular in estimating whether an acaricidal treatment would be able to reduce the risk of transmission of a pathogen. Knowing the SOT of a pathogen by a tick species as well as the speed of kill of a parasitic drug can facilitate further research on the potential for disease prevention with such a drug [1, 20, 22, 25].

This study aims to assess the SOT of major canine TBPs, including *E. canis* by laboratory-infected *R. sanguineus*, *A. phagocytophilum* by laboratory-infected *I. ricinus*, and *B. burgdorferi* sensu stricto (s.s.) by laboratory-infected *I. ricinus*, using the same continuous-flow in vitro feeding system (CFIFS). Having a standardized experimental design will allow us to test and compare several tick/pathogen complexes.

## Methods

### Study design

**Fig. 1:**
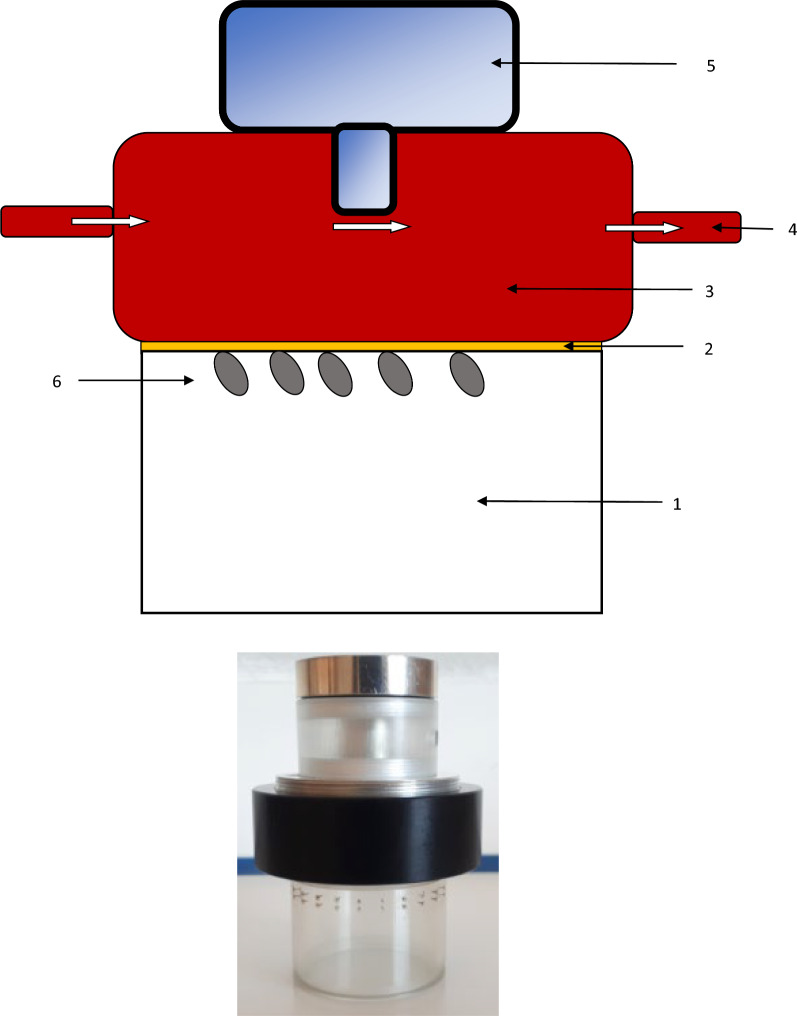
Feeding device with continuous blood flow: 1: tick chamber; 2: silicone membrane; 3: blood chamber; 4: blood flow; 5: heating block with heating element extending downward into the blood chamber; 6: ticks attached to the silicone membrane and feeding

Two experiments were conducted to investigate the SOT of pathogens by ticks, using an adapted version of the United States Department of Agriculture (USDA)-developed CFIFS [26] (Tables 1, 2, 3). Each study used a single CFIFS consisting of four feeding devices (FDs), each with its own feeding media (whole blood collected on experimental animals of the research center, cattle and dogs), for a total of four to six replicates per model (Fig. 1). These experiments were conducted between 2021 and 2023.

**Table 1:** Summary of the similarities and differences between the two experimental designs

	Experimental design 1a and b	Experimental design 2
Pathogen and vector	*Ehrlichia canis*/*Rhipicephalus sanguineus* *Anaplasma phagocytophilum*/*Ixodes ricinus*	*Borrelia burgdorferi* s.s./*Ixodes ricinus*
Blood media	Bovine blood	Canine blood
Replenishment of blood (media)	Every 6 h	Group 1: First 6 h, then every 12 h Group 2: Every 12 h
Sampling	1 ml of blood every 3 h	1 ml of blood every 3 h Swab sample on the inside face of the membrane every 6 h during the blood change time points
Sampling duration	Up to 72 h	Up to 72 h
Quantitative PCR (qPCR) analysis	On pooled samples	On individual samples
Total assessment time points	24 for tick assessment (attachment and viability) and qPCR 1 for tick weight	24 for tick assessment (attachment and viability) and qPCR 1 for tick weight 12 for blood swab qPCR

**Table 2:** The vector-borne pathogens, tick vectors, and their infection ratios in the studies

Experiment	Pathogen	Vector	Infection ratio %	Reference method for determining the tick infection ratio
1a	*E. canis*	*R. sanguineus*	18.3	[27]
1b	*A. phagocytophilum*	*I. ricinus*	56	[28]
2	*B. burgdorferi *s.s.	*I. ricinus*	76	In-house PCR assay

**Table 3:** In vitro system specifications

Experiment	1a and b		2
Tick species	*Rhipicephalus sanguineus*	*Ixodes ricinus*	*Ixodes ricinus*
Pathogen carried	*Ehrlichia canis*	*Anaplasma phagocytophilum*	*Borrelia burgdorferi* s.s.
Membrane			
Thickness	0.06–0.08 mm	0.01–0.19 mm	0.10–0.19 mm
Kairomones	Dog hair	Dog hair	Dog hair
Blood			
Host species	Cattle	Cattle	Dog
Gentamicin	10 mg/ml	10 mg/ml	10 mg/ml
Glucose	2 g/l	2 g/l	2 g/l
Number of adult ticks per feeding device (FD)	100 (50 F/50 M)	60 (50 F/10 M)	58 (48 F/10 M)
Replicates (each FD is a replicate)	4	4	6 2 groups, each with 3 FDs
Tick pre-activation			
Time	7 days^a^	5 days	0–5 days^b^
Temperature	26 °C	22 °C	22 °C
Relative humidity	75–85%	90%	90%
Photoperiod	16 h light; 8 h dark	14 h light; 10 h dark	14 h light; 10 h dark
FD incubation			
Temperature	27–29 °C	37 °C	37 °C
Relative humidity	60–70%	90–95%	90–95%
Photoperiod	24 h dark	24 h dark	24 h dark

F: female; M: male

^a^Activation period could vary between 48 h and 7 days, depending on the age of the ticks

^b^Dependent on tick viability when received

Experiment 1 examined the SOT of *E. canis* transmitted by *R. sanguineus* and *A. phagocytophilum* by *I. ricinus*.

Experiment 2 investigated the SOT of *B. burgdorferi* s.s. by *I. ricinus.* Experiment 2 was conducted after a preliminary study (unpublished) with infected wild-caught *Ixodes* ticks from the Netherlands, which was unable to demonstrate transmission. Thus, the methodology was modified for the second experiment to use artificially infected laboratory-reared ticks.

The details, similarities, and differences between the experimental designs are summarized in Tables 1 and 3. All infected adult ticks were obtained by feeding the nymphal stages on infected hosts. The adult ticks were all used 2–4 months after molting.

### Ticks and vector-borne pathogens

#### *Rhipicephalus sanguineus*

In experiment 1a, 100 laboratory-bred adult ticks infected with a European strain of *E. canis* with an equal sex ratio were used in each of the four feeders. The tick strain originated from Carros, France, whereas the *E. canis* was isolated from a dog in South Africa, as detailed in a previous work [3]. Before being introduced into the feeders, the ticks were taken from a conservation insectary (low temperature and darkness) and underwent an activation period of a few days in the insectary with controlled temperature, humidity, and photoperiod (Table 1). Infected nymph ticks were obtained by infestation on an infected donor dog (i.e., blood smear, PCR and serologically positive to *E. canis* infection). Subsequently, adult-infected *R. sanguineus* ticks were obtained after the molting of the collected engorged nymphs.

#### *Ixodes ricinus*

In experiment 1b, 60 *I. ricinus* ticks (50 females and 10 males) were used in each of the four feeders. The ticks were bred in a laboratory at Utrecht University, originating from their initial collection in the Netherlands. The ticks were infected by feeding as nymphs on a dog infected with the *A. phagocytophilum* strain (TIBA strain), confirmed by PCR and serology from dog blood. The *A. phagocytophilum* strain used (TIBA strain) was isolated in June 2015 from a clinical case (dog) in Terschelling, the Netherlands [29]. Since that time, the strain has been maintained by passages between ticks and sheep, rabbits, or dogs.

In all research activities, dog blood infected by either *A. phagocytophilum* or *E. canis* was stored at −80 °C with 10% dimethyl sulfoxide (DMSO) as a cryoprotectant.

In experiment 2, 58 *I. ricinus* ticks (48 females and 10 males) were used in each of the six feeders. These adult ticks were fed as nymphs on mice infected with *B. burgdorferi* s.s. The infection in mice was confirmed by PCR.

The infection rate of the tick batches used in the experiments is given in Table 2.

### Preparation of the in vitro system

#### Preparation of the feeding membranes

The feeding membranes of the FDs were prepared by treating commercial Goldbeater's skin with 5 g each of Ecoflex Supersoft 00-50 silicone A and B (Smooth-On, Easton, PA), mixed with 2 ml of hexane (Sigma-Aldrich). Goldbeater's skin is the processed outer membrane of the intestine of cattle (the gut is soaked in a dilute solution of potassium hydroxide, washed, stretched, beaten flat and thin, and treated chemically to prevent putrefaction). The silicone was subsequently rolled manually over the skin to ensure homogeneity. The membrane was polymerized for at least 12 h before being used within 5 days. Its thickness was adapted to each tick species.

Dog hairs were added to the membrane to stimulate tick feeding.

#### Preparation of the blood media and sampling

In experiment 1, parasite-free bovine blood was collected from one experimental cow using blood collection bags supplemented with citrate phosphate dextrose (CPD) and stored at 4 °C. Blood not older than 2 weeks was used for tick feeding. All procedures, including adding and changing of blood, were performed in a biosafety cabinet to avoid any contamination. All parts of the FD were sterilized before use. Approximately 10 ml of blood was preheated to 37 °C in a water bath and used in each FD, supplemented with gentamicin and glucose.

In experiment 2, parasite-free canine blood was collected from donor dogs (experimental Beagle dogs) not treated with any acaricide using blood heparin collection tubes and stored at 4 °C until use. Blood not older than 2 weeks was used for tick feeding. Table 3 contains the details of the in vitro specifications.

In experiment 1a and b, blood was replenished every 12 h. In experiment 2, the blood was changed for group 1 after 6 h and subsequently every 12 h, whereas the blood was changed for group 2 every 12 h. The goal of alternating between two groups in experiment 2 was to increase the number of (blood swab) collection time points to every 6 h. Thus, the blood replenishment points (h) in group 1 occurred at 0 (baseline), 6, 18, 30, 42, 54, and 66 h, and for group 2 at 0 (baseline), 12, 24, 36, 48, 60, and 72 h.

A swab sample was taken from the inner surface of the feeding membrane during blood change time points in experiment 2. Two membrane swabs (using medical sterilized cotton swabs) were taken from the inside of the membrane to collect a maximum of blood attached to the membrane. Collection was performed during a blood change procedure from each of the six feeding chambers (three feeders per group) at 6, 18, 30, 42, 54, and 66 h for group 1, and at 12, 24, 36, 48, 60, and 72 h for group 2. The swabbing objective was to determine whether the *Borrelia* spirochetes remained attached to the inner silicone membrane after inoculation by the adult ticks before entering the bloodstream.

In all experiments, 1 ml of blood was sampled from the CFIFS every 3 h to detect the pathogen transmission by quantitative PCR (qPCR). At each sampling, the FD was replenished with the same volume of clean blood to ensure consistency in the total blood volume in the system.

Following the collection of blood from the FD for pathogen screening, the membrane was rinsed with sterile, heated (37 °C) physiological water to remove any residual blood.

### Tick attachment and viability assessments

Tick attachment and vitality assessments were performed at 3-h intervals, and no ticks were removed during the 72-h period of the experiment. The ticks were all unfed at the time of introduction into the system and were removed at the end of the 72 h. Following that, they were counted and categorized based on the criteria in Table 4.

**Table 4:** Tick assessment categories

Attachment status	Sex	Viability
Free	Male	Live
Free	Male	Dead
Free	Female	Live
Free	Female	Dead
Attached	Male	Live
Attached	Male	Dead
Attached	Female	Live
Attached	Female	Dead

Tick weights were measured before for all ticks and after tick feeding for attached ticks (individually and by sex, except in experiment 2, where only females were weighed) and used to confirm their infection status by qPCR.

### qPCR analysis

Blood samples collected from the in vitro feeders of the study were frozen at −70 °C prior to DNA extraction. DNA was extracted from whole blood using the NucleoSpin^®^ Blood kit (Macherey–Nagel, Düren, Germany). DNA was then analyzed by qPCR at the end of the experiments [3, 29]. The qPCR methods were described in previously published studies on *E. canis* [3], *A. phagocytophilum* [29], and *B. burgdorferi* s.s. (in-house qPCR method).

The PCR limit of detection (organisms/ml of blood) for each species was as follows: *E. canis* ≥ 250; *A. phagocytophilum* ≥ 19, and *B. burgdorferi* s.s. ≥ 125 [3, 29]. The qPCR analysis for experiment 1a and b was performed on pooled blood samples taken at the same time points from all four feeders to reduce the number of qPCRs. In experiment 2, to increase the sensitivity of detection after a preliminary experiment with no detection (unpublished), qPCRs were performed on individual blood samples from each of the six feeders.

A blood sample was considered infected if it tested positive for the pathogen by qPCR analysis.

### Data analysis

Descriptive statistics were used to determine the minimum SOT time from the start of tick feeding in each feeder, as well as the average transmission time for each tick species across the feeders. Descriptive statistics (mean, minimum, maximum, standard deviation (SD), coefficient of variation (CV), geometric mean, and median) for tick counts and tick weight were computed and tabulated for each time point.

## Results

### Tick attachment and viability assessments

#### *Rhipicephalus sanguineus* infected with *Ehrlichia canis*

Table 5 provides a summary of the results. Tick attachment was observed on all four feeders 3 h after tick introduction, with a mean of 3.5% of attached ticks. Mean attachment rate corresponds to the average of the four feeders at a given time point. Attachment increased every 3 h to 89.7% at 57 h. Thereafter, the number of attached ticks dropped slightly until 72 h, with a mean percentage of attachment of 84.4% at 72 h.

**Table 5:** Summary of results

Tick species	Parasites	Time point			First detection time point blood sample	First detection time point swab sample	% of live attached ticks (mean) at the first detection time point
		First tick attachment	Time for max attachment of ticks	% of ticks attached at max attachment (mean)			
*R. sanguineus*	*E. canis*	3 h	57 h	89.7	9 h	–	37.5
*I. ricinus*	*A. phagocytophilum*	3 h	51 h	20.5	18 h	–	12.5
*I. ricinus*	*B. burgdorferi*	3 h	18 h	30.2	48 h	6	16.3

The first detection point is the time when the qPCR becomes positive

The SD appeared to be highest at the first time point, and it gradually decreased at each subsequent time point until it was close to zero after 48 h. The CV was higher at the beginning of the experiment but decreased to less than 10% after 48 h.

**Fig. 2:**
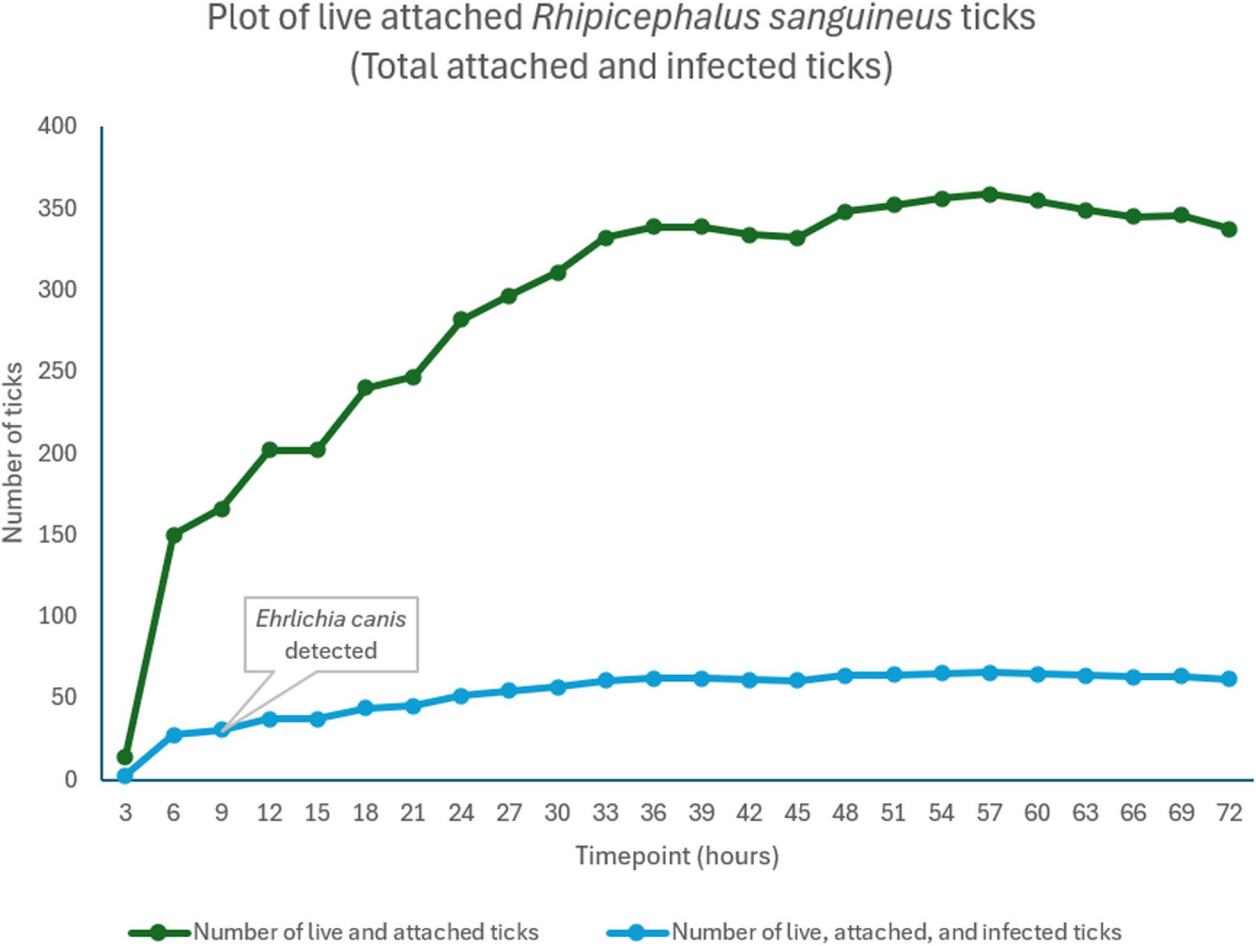
Plot of live attached *R. sanguineus* total and estimated infected ticks. There were 400 ticks in the four feeding chambers. The infection ratio of *E. canis* was 18.3%. Total number of live and attached ticks = 400 × percentage of tick attachment. Light blue = estimated number of live, attached, and infected female ticks = total number of live and attached female ticks × 18.3%

Given the 18.3% infection ratio of *E. canis* in *R. sanguineus* ticks, the estimated average of attached infected ticks was approximately 2–3 ticks from all live attached ticks from the four feeders (400 ticks) at 3 h, 27–28 ticks at 6 h, 36–37 ticks at 12 h, and a total maximum of approximately 65 ticks at 57 h (Fig. 2).

The system shows a low mortality ratio of free and attached *R. sanguineus* ticks, with a maximum mean percentage of 13.3% at 72 h. Mortality was nearly 0% at the start and gradually increased, reaching a mean of 2.8% 24 h after incubation and 7.3% 48 h later. The four FDs had comparable mortality rates, with SD ranging from 1.29 to 6.45%.

#### *Ixodes ricinus* infected with *A. phagocytophilum*

After introducing 50 female ticks and 10 males in each feeder, attachment was observed in all four feeders 3 h after introduction (2–14% live attached ticks). Attachment increased every 3 h until 51 h (4–30% tick attachment), and some feeders dropped off until 72 h (8–26% tick attachment). Tick attachment ratios ranged from 5% at 3 h post-infestation to 20.5% after 51 h.

The SD remained constant, peaking at 21 h before decreasing. The CV increased at the first time point and then decreased, but it remained above 50%.

**Fig. 3:**
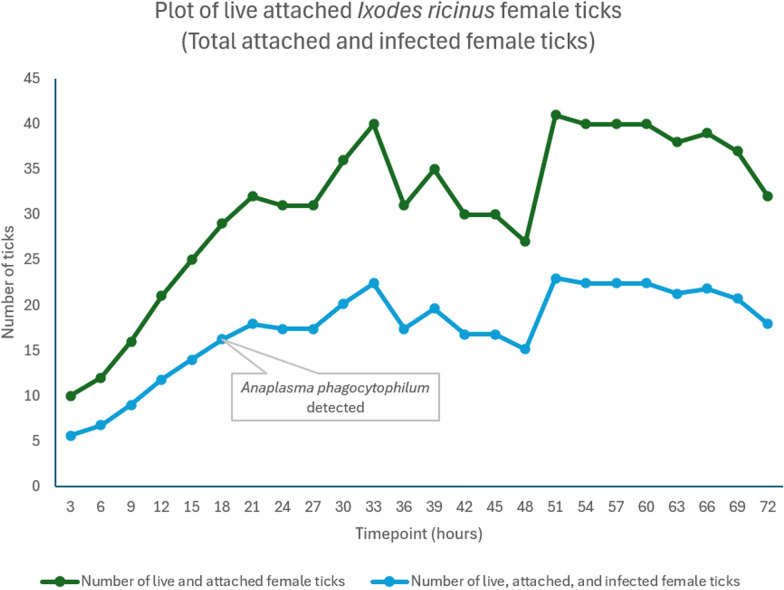
Plot of live attached *I. ricinus* total and estimated infected ticks. There were 200 female ticks in the four feeding chambers. The infection ratio of *A. phagocytophilum* was 56%. Total number of live and attached female ticks = 200 × percentage of tick attachment. Light blue = estimated number of live, attached, and infected female ticks = total number of live and attached female ticks × 56%

Considering the 56% infection ratio of *A. phagocytophilum* in *I. ricinus*, the estimated average of attached infected ticks was approximately 5–6 infected ticks from all live attached ticks from the total infested female ticks in four feeders (200 ticks) at 3 h, 11–12 ticks at 12 h, and a total maximum of approximately 22 live female infected ticks at 33 h (Fig. 3).

The initial phase of mortality was low, with dead ticks appearing at 9 h (mean mortality percentage of 1.5%), followed by higher mortality at 48 h (mean mortality percentage of 31.5%), and a maximum of 42.5% at 72 h.

**Fig. 4:**
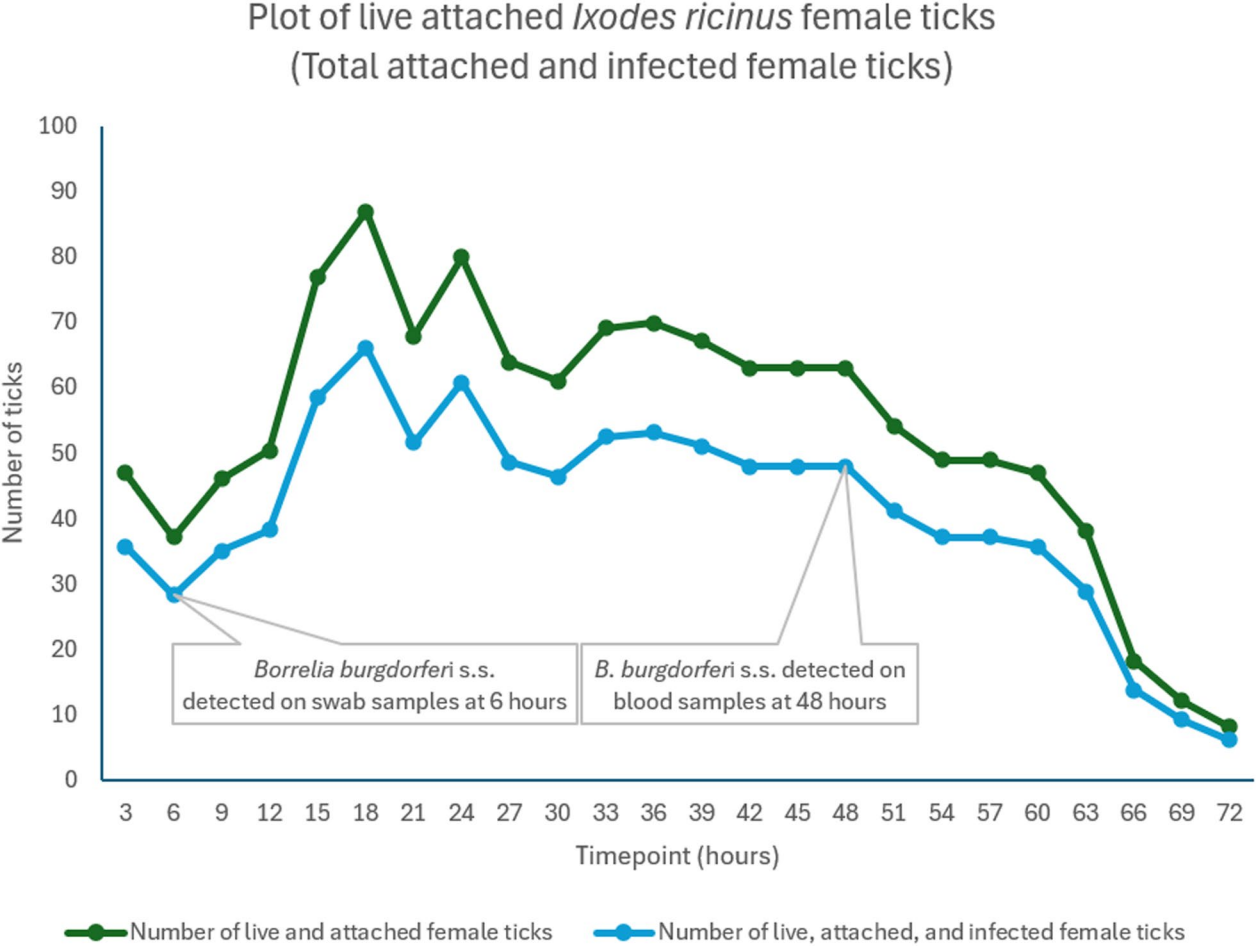
Plot of live attached *I. ricinus* total and estimated infected ticks. There were 288 female ticks in the six feeding chambers. The infection ratio of *B. burgdorferi* was 76%. Total number of live and attached female ticks = 288 × percentage of tick attachment. Light blue = estimated number of live, attached, and infected female ticks = total number of live and attached female ticks × 76%

FD 2 had the lowest mortality rate throughout the experiment, reaching a maximum of 34% at 72 h. In contrast, FDs 1 and 4 had the highest mortality rates, which peaked at 48% and 50%, respectively, at 72 h. Mortality in FD 3 decreased from 18% to 16% at 15 h, primarily due to inaccurate mortality estimation at the start (Fig. 4).

#### *Ixodes ricinus* infected with* B. burgdorferi* s.s.

After the introduction of 48 females and 10 males, the female *I. ricinus* ticks attached in each of the six feeders 3 h after tick introduction (8.3–29.2% live attached ticks), and attachment increased every 3 h of assessment until all FDs had a percentage of attachment ranging from 10.4% to 41.7% at 24 h and 6.3% to 47.9% at 48 h, after which it decreased, ranging from 0% to 8.3% at 72 h. The minimum tick attachment ratio was 0% in FDs 2 and 3, measured 72 h after tick introduction.

The SD ranged between 2.64% and 15.41%, with the highest value recorded at 48 h. The CV ranged between 40% and 70% at the beginning of the experiment, but it began to increase during the final time point, peaking at 72 h.

Based on a 76% infection ratio of *B. burgdorferi* s.s. in *I. ricinus*, the average estimated number of attached infected ticks in six feeders (288 ticks) was 36 at 3 h, 28 at 6 h, and 66 at 18 h.

Mortality was low at the first time points. Dead ticks were first seen at 9 h (2.1% in FD 2), and it was lower for the first 24 h (mean mortality percentage of 11.8%) in comparison with the other time points. The maximum mean percentage of mortality at 72 h was 95.1%. During the experiment, FDs 1 and 6 showed the lowest mortality value, and FDs 3 and 5 had the highest mortality. At the last time point, the mortality increased significantly, reaching a mortality rate of approximately 89–100% in the FDs.

### Tick weight gain after incubation

The differences in average individual tick weight after 72 h were calculated. The mean weight gain for attached *R. sanguineus* male and female ticks was 19% (mean weight from 291 to 346 mg) and 19.7% (mean weight from 275 to 330 mg), respectively. In experiment 1b, female *I. ricinus* ticks gained 21% (mean weight from 174 to 210 mg) of their body weight, while male ticks lost 7.4% (mean weight from 87 to 81 mg), indicating they were not feeding.

In experiment 2, males were not weighed, and the difference between the mean weight of female ticks of the six FDs after the 72 h incubation showed that the mean weight increased from 96.18 mg (SD = 9.99) to 104.15 mg (SD = 8.19), indicating a weight gain of 8.3%.

### qPCR results

#### Detection of *E. canis*

Blood sampled from the four FDs was pooled by time point. *Ehrlichia canis* was detected by PCR at 9 h after tick introduction in the feeding system, i.e., 6 h after first tick attachments.

#### Detection of *A. phagocytophilum*

Blood sampled from the four FDs was pooled by time point. *Anaplasma phagocytophilum* was detected by PCR at 18 h after tick introduction in the system, i.e., 15 h after first tick attachments.

#### Detection of *B. burgdorferi*

Blood samples from the six FDs were analyzed individually per time point and per FD. *Borrelia burgdorferi* was detected by PCR only in FD 5 at 48 h after tick introduction in the system, i.e., 45 h after first tick attachments.

Swab samples collected at each time point detected B*. burgdorferi* in FDs 3 and 5 at 6 and 12 h after tick introduction in the system, respectively, i.e., 3 h after first tick attachments.

## Discussion

### Validation of the in vitro model

In vitro methods can improve the understanding of the vector–pathogen relationship and vector feeding behavior before the use of animals [20, 25]. They also enable a controlled assessment of VBP transmission times.

*Rhipicephalus sanguineus* infected with *E. canis* demonstrated a large initial SD that decreased significantly over time, indicating that the four FDs were equivalent. After 24 h, the CV began to decrease, indicating high model homogeneity. Mortality rates were lowest at the start of the experiment, with most dead ticks found at later time points.

For *I. ricinus* infected by *A. phagocytophilum*, low initial attachment rates affected the SD and CV. Although mortality rates were higher than for *R. sanguineus*, they did not exceed 50%, with a CV of 17.2%, making it acceptable to follow the feeding for 72 h.

In the case of *I. ricinus* infected with *B. burgdorferi *s.s., the SD remained low, never exceeding 15%. We can conclude that all six FDs were equivalent. The CV for female tick attachment varied around 50% but increased near the end of the experiment. The highest attachment percentage was observed for FD 3 in group 1 and FD 5 in group 2, which are the two FDs where *Borrelia* s.s. transmission was found. Mortality rates were low during the initial time points, increasing 66 h after tick introduction. Although the mortality rate was high, the fact that mortality occurred late in the experiment allowed us to study the feeding of ticks during at least the first 2 days. The mortality rates were very high at 72 h, which could be attributed to the high infection ratio and/or laboratory conditions. According to Benelli, *Borrelia* spp.-infected ticks may have a higher mortality rate and also exhibit behavioral changes that may aid in pathogen transmission, such as altered questing behavior [30].

Weight gain statistics for both *I. ricinus* experiments showed that the female ticks successfully ingested blood while attached to the silicone membrane. Although the ticks were still in the slow feeding phase with limited uptake, they were able to transmit *Borrelia* spp. Male *Ixodes* were not attaching or feeding in these experiments.

### Determining the speed of transmission

Blood samples were collected every 3 h after the feeding system was initiated to be able to precisely determine the SOT in relation to the first attachment time of the ticks. When pathogens were detected using qPCR, two transmission times were considered: (1) the interval between the start time and qPCR positive time, and (2) the interval between attachment time and qPCR positive time (±3 h)—referred to as the minimum SOT.

The “start time” refers to the time when ticks were introduced into the system. The “attachment time” refers to the first time the ticks were observed to be attached. As the qPCR and the observation of tick attachment were conducted every 3 h, it is not possible to give an exact time of transmission, but it can be estimated that the first transmission occurred during the 3 h before its first detection.

Fourie et al. previously noted that transmission does not mean infection [29]. In this study, the transmission time was assessed without taking any quantity of pathogens into account. The minimum infective dose is not known in dogs for these pathogens. It may also vary based on the pathogenicity of each strain, the individual receptivity of the host itself, and probably associated factors related to the tick vectors (facilitator effects of the saliva). The present in vitro study did not provide any information with quantitative data, as it may not be equivalent to the in vivo situation. Therefore, only the first detection of the pathogen in blood was analyzed.

#### *Ehrlichia canis*

*Ehrlichia canis* was detected 6 h after attachment. As a result, the minimum SOT ranged from 3 to 6 h after tick attachment. At 3 h, 3.5% of ticks were already attached, and at 6 h, an average of 27 infected ticks were estimated from an average of 37.5% live attached ticks. The results are consistent with a previous study that found *E. canis* DNA in nutritive blood medium as early as 8 h after tick introduction in a feeding system and *E. canis* transmission within 3 h in dogs [3].

Thus, to reduce the risk of *E. canis* transmission, acaricides that repel, block-feeding, or kill ticks quickly should act very rapidly [3]. Such protection has only been demonstrated in experimental clinical studies in dogs treated with topical ectoparasiticides containing the repellent permethrin [25, 31]. The repellent effect of permethrin is an anti-feeding effect, which explains the possibility of reducing the risk of transmission of *E. canis*.

#### *Anaplasma phagocytophilum*

*Anaplasma phagocytophilum* was detected 15 h after the attachment started. Attachment occurred 3 h after tick introduction. Therefore, the SOT is estimated to be between 12 and 15 h after tick attachment. The *A. phagocytophilum* infection rate of 56% in *I. ricinus* ticks compensated the low number of attached ticks.

Previous studies indicated that ticks must attach for 36–48 h to transmit *A. phagocytophilum* [32, 33]. Another study demonstrated the presence of *A. phagocytophilum* DNA in blood samples from artificial feeders where infected *I. ricinus* ticks had fed for only 6 h [29]. In a study with dogs, infection took 48 h [29], suggesting that the establishment of infections in dogs is dependent on both the SOT and the minimum inoculation dose.

To prevent the spread of *A. phagocytophilum*, it seems that acaricidal products should repel ticks or kill them in a few hours. Such protection has been demonstrated in experimental clinical studies in dogs treated with topical ectoparasiticides containing repellent permethrin [25].

#### *Borrelia burgdorferi* s.s.

*Borrelia burgdorferi* was detected in swab samples within 3 to 6 h and blood samples within 42 to 45 h after tick attachment started. It has been demonstrated that transmission can be prevented in dogs with acaricidal products containing either afoxolaner or sarolaner [25], which kill ticks within 48 h. This either confirms the importance of the infective dose inoculated, or indicates that these acaricidal molecules blocked the natural feeding behavior.

In previously published studies, *B. burgdorferi* spirochetes were transmitted by infected ixodid ticks 48–72 h after tick attachment to the host [34, 35], but others have reported 24–48 h [18, 23, 36, 37]. The swab findings support the hypothesis that spirochetes can remain on the animal's skin following tick inoculation before entering the bloodstream [38–40].

Some authors have indicated that *Borrelia* spp. can be transmitted in less than 16 h or less than 24 h [13, 18], but it is most common after ≥ 60 h of nymphal attachment [37]. In one study, a hamster became infected with *B. burgdorferi* when exposed to infected *Ixodes dammini* ticks for 24 h [38]. In another study, *B. burgdorferi* was not transmitted to mice within the first 24 h of exposure to infected *Ixodes scapularis* ticks [34].

It was hypothesized by researchers that pathogen transmission takes longer for *B. burgdorferi* than for *Ehrlichia* and *Anaplasma* due to a pre-activation period [22, 38]. Several factors may influence this bacterial pre-activation time [22, 35], including the start of blood ingestion by the ticks [39–41]. *Borrelia burgdorferi* spirochetes have been found in tick salivary glands and other organs before and at the onset of feeding [40]. They could be inoculated into the host during the first phase of tick feeding, before the engorgement phase [18].

Longer tick attachment times were associated with an increased risk of infection [36, 38]. Nymphs may transmit more quickly than adult ticks [13, 18]. In addition to the stage of the tick, the host should also play a role in the SOT. Transmission seems quicker in rodents than in larger mammals. In one study, *B. burgdorferi*-infected *I. ricinus* nymphs were allowed to feed on gerbils for brief periods. Within 17 h of tick attachment, 50% of gerbils became infected. After 48 h of feeding, all gerbils developed *Borrelia* infections [41].

It seems that transmission in dogs takes more time than in other models such as rodents.

### Tracking transmission

CVBDs have a significant clinical and public health impact, particularly affecting dogs in tropical and subtropical regions, primarily in middle- and low-income countries [25]. Preventing CVBDs is crucial for dog health and welfare, as well as reducing the zoonotic risk to humans [1, 25].

The most common CVBDs, such as babesiosis, ehrlichiosis, leishmaniosis, and dirofilariosis, are typically targeted through a combination of strategies, including ectoparasiticides for vector control, therapeutic treatments for infected animals, chemoprophylaxis, and to a lesser extent, vaccination [25].

Acaricides can be used to repel, disrupt feeding, or quickly kill arthropod vectors, which reduces pathogen transmission. Theoretically, VBP transmission can be prevented by impeding arthropod feeding or killing arthropod vectors before pathogens are transmitted to the hosts [20].

The efficacy of any product in blocking VBP transmission, however, is also dependent on the pathogens’ transmission times; quickly transmitted pathogens such as *E. canis* (transmitted by *R. sanguineus* s.l. in about 3 h, in vivo experiment) [3] seem better targeted by synthetic pyrethroids used topically and providing an anti-feeding effect [42]. Nevertheless, systemic acaricides like isoxazolines have demonstrated their ability to prevent the transmission of *B. burgdorferi* s.s. and *Babesia canis*. This may be related to a longer time for transmission, but also to the possibility of these drugs blocking the feeding behavior before killing the ticks [31].

### Limitations of the in vitro study

The use of bovine blood may have affected the minimum SOT in experiment 1, because the blood may be less compatible to allow bacterial multiplication in the blood, or less attractive for some tick species. Nevertheless, cattle are a natural host for *I. ricinus*, and *A. phagocytophilum* is a common infection in cattle [15].

The use of gentamicin (a broad-spectrum antibiotic) in experiment 1 to limit the contamination and development of Gram-negative bacteria required for proper blood storage may have impacted the results and the rate of detection of *A. phagocytophilum*.

While in vitro studies using artificial feeding systems offer preliminary insights into vector–pathogen interactions, feeding behavior, and transmission times, there are some limitations. An example is the lack of host immune responses in artificial feeders, which can interfere with feeding and VBP transmission [21].

The success of in vitro feeding systems is determined by arthropod feeding rates, which are also influenced by tick mouthpart size and feeding behavior (for example, *Ixodes* spp. feed faster than *Rhipicephalus* spp.) [3, 24, 29]. Some ticks may feed less frequently in an in vitro feeder than in live hosts [29], and salivary secretion dynamics and feeding mechanics may differ in vitro [35, 36].

While in vitro studies can provide useful initial data, complementing in vitro findings with in vivo animal studies remains essential to fully assess the preventive efficacy of parasiticides in preventing pathogen transmission. In vivo animal studies may also add the dimension of disease transmission, in addition to pathogen transmission. Nevertheless, in vivo studies should be limited as much as possible due to ethical concerns, feasibility, and cost.

## Conclusions

The SOTs of three pathogen species, i.e. *E. canis* (3–6 h), *A. phagocytophilum* (12–15 h), and *B. burgdorferi* s.s. (42–45 h in blood, 3–6 h on membrane), were determined using a CFIFS. A key benefit of such a standardized feeding system is the ability to compare pathogens/ticks and classify the SOT amongst the pathogens. The results of this study clarify the transmission biology of the vector–parasite model, and may be helpful in developing transmission-blocking compounds for companion animals. Other pathogens, especially *Babesia* spp., should be studied with this system.

## Data Availability

No datasets were generated or analyzed during the current study.
